# Supersaturation-controlled microcrystallization and visualization analysis for serial femtosecond crystallography

**DOI:** 10.1038/s41598-018-20899-9

**Published:** 2018-02-07

**Authors:** Dan Bi Lee, Jong-Min Kim, Jong Hyeon Seok, Ji-Hye Lee, Jae Deok Jo, Ji Young Mun, Chelsie Conrad, Jesse Coe, Garrett Nelson, Brenda Hogue, Thomas A. White, Nadia Zatsepin, Uwe Weierstall, Anton Barty, Henry Chapman, Petra Fromme, John Spence, Mi Sook Chung, Chang-Hyun Oh, Kyung Hyun Kim

**Affiliations:** 1 Department of Biotechnology & Bioinformatics, Korea University, Sejong, Korea; 2 Department of Electronics & Information Engineering, Korea University, Sejong, Korea; 3 Department of Structure and Function of Neural Network, Korea Brain Research Institute, Daegu, Korea; 4 Department of Chemistry, Arizona State University, Tempe, Arizona USA; 5 Department of Physics, Arizona State University, Tempe, Arizona USA; 6 Biodesign Center for Applied Structural Discovery, Arizona State University, Tempe, Arizona USA; 7 Center for Free-Electron Laser Science, Deutsches Elektronen-Synchrotron DESY, Hamburg, Germany; 8 Department of Food and Nutrition, Duksung Women’s University, Seoul, Korea

## Abstract

Time-resolved serial femtosecond crystallography with X-ray free electron laser (XFEL) holds the potential to view fast reactions occurring at near-physiological temperature. However, production and characterization of homogeneous micron-sized protein crystals at high density remain a bottleneck, due to the lack of the necessary equipments in ordinary laboratories. We describe here supersaturation-controlled microcrystallization and visualization and analysis tools that can be easily used in any laboratory. The microcrystallization conditions of the influenza virus hemagglutinin were initially obtained with low reproducibility, which was improved by employing a rapid evaporation of hanging drops. Supersaturation-controlled microcrystallization was then developed in a vapor diffusion mode, where supersaturation was induced by evaporation in hanging drops sequentially for durations ranging from 30 sec to 3 min, depending on the protein. It was applied successfully to the microcrystal formation of lysozyme, ferritin and hemagglutinin with high density. Moreover, visualization and analysis tools were developed to characterize the microcrystals observed by light microscopy. The size and density distributions of microcrystals analyzed by the tools were found to be consistent with the results of manual analysis, further validated by high-resolution microscopic analyses. Our supersaturation-controlled microcrystallization and visualization and analysis tools will provide universal access to successful XFEL studies.

## Introduction

Serial femtosecond crystallography (SFX) using X-ray free electron laser (XFEL) sources opened a new avenue to time-resolved structural determination of reaction intermediates^1–4^. A major obstacle to synchrotron-based experiments is the dose limit of the X-ray light sources to avoid damage to nano- to micron-sized crystals. The frequent requirement of using frozen samples can bias the structural ensembles in protein crystals and limit our ability to study protein motions at near-physiological temperature^5,6^. Ultra-intense and ultra-fast X-ray pulses from XFELs are used to overcome these problems, mainly based on the diffraction-before-destruction mode of data collection, in studies including the determination of atomic structures of membrane proteins and *in vivo* grown crystals, the early stages of photochemical reactions, and conformational changes on microsecond timescales^5,7–9^. Although XFELs offer extremely intense X-ray beams and room temperature data collection, their application is limited because a large number of highly ordered nano- to micron-sized crystals is essential for successful XFEL experiments at room temperature. However, growth and characterization of high-quality nanocrystals remain bottlenecks, because many ordinary laboratories lack the necessary equipments.

Microcrystals are obtained by mixing a protein with precipitant solutions in a batch method, for which a microcrystallization phase diagram is needed to produce massive nucleation^10^. A precipitant solution is added dropwise to a protein solution in a free interface diffusion method, allowing for a high nucleation rate at the protein/precipitant interface^10^. Vapor diffusion is still the most commonly employed method for microcrystallization, and cell-based *in vivo* crystallization has been reported^11^. Lipidic cubic phase (LCP) microcrystallization of membrane proteins was demonstrated in XFEL experiments, and the LCP was also used as a suitable carrier medium for microcrystals of soluble proteins^12–14^. However, nano- or microcrystal growth methods have remained largely unexplored compared to those for single crystal growth for synchrotron X-ray diffraction studies.

Monitoring the size of microcrystals is another major bottleneck, despite increasing use of bright-field high-resolution microscopy, dynamic light scattering (DLS), birefringence microscopy, intrinsic UV fluorescence imaging, second-order nonlinear imaging of chiral crystals (SONICC), X-ray powder diffraction, and transmission electron microscopy (TEM), which are not easily available in many ordinary laboratories^15–19^. The most important characteristics of nanocrystals are the size, quality and crystalline order. The size limitation ranges from 200 nm to 2 μm and is extended to 30–50 μm when LCP and nanoflow electrospinning injectors with a reduced flow rate and lower sample consumption are used^20,21^. Nevertheless, detecting nano- to micron-sized crystals smaller than 50 μm and distinguishing between amorphous aggregates and crystalline protein samples are not easy tasks, when only light microscopy is available. In addition, optimization of the crystal density and homogeneity in large sample volumes is needed for complete SFX data collection to increase the hit rate and prevent liquid nozzle clogging^22^. Growth and characterization of nano- to micron-sized crystals, which were previously seen as of no value for single crystal data collection in synchrotrons, therefore become an important task in XFEL-based structural studies.

In this study, microcrystallization of the recombinant influenza virus hemagglutinin (HA), which was not very reproducible, was initially examined. To overcome the low reproducibility of HA microcrystallization, we assessed rapid evaporation from hanging drops, in which protein precipitates started to appear at the first coverslip, and the coverslips were flipped over to seal the wells, producing HA microcrystals with improved reproducibility. A supersaturation-controlled microcrystallization method was then developed to control solvent evaporation in hanging drops sequentially for durations ranging from 30 sec to 3 min, depending on the protein. It was successfully applied to rapid formation of microcrystals of lysozyme, ferritin and HA at high density. Moreover, nanocrystal image visualization and analysis tools were developed to monitor and characterize the crystal density and size distributions in drops using images obtained by a light microscope.

## Results

### Microcrystallization of HA

**Figure 1:**
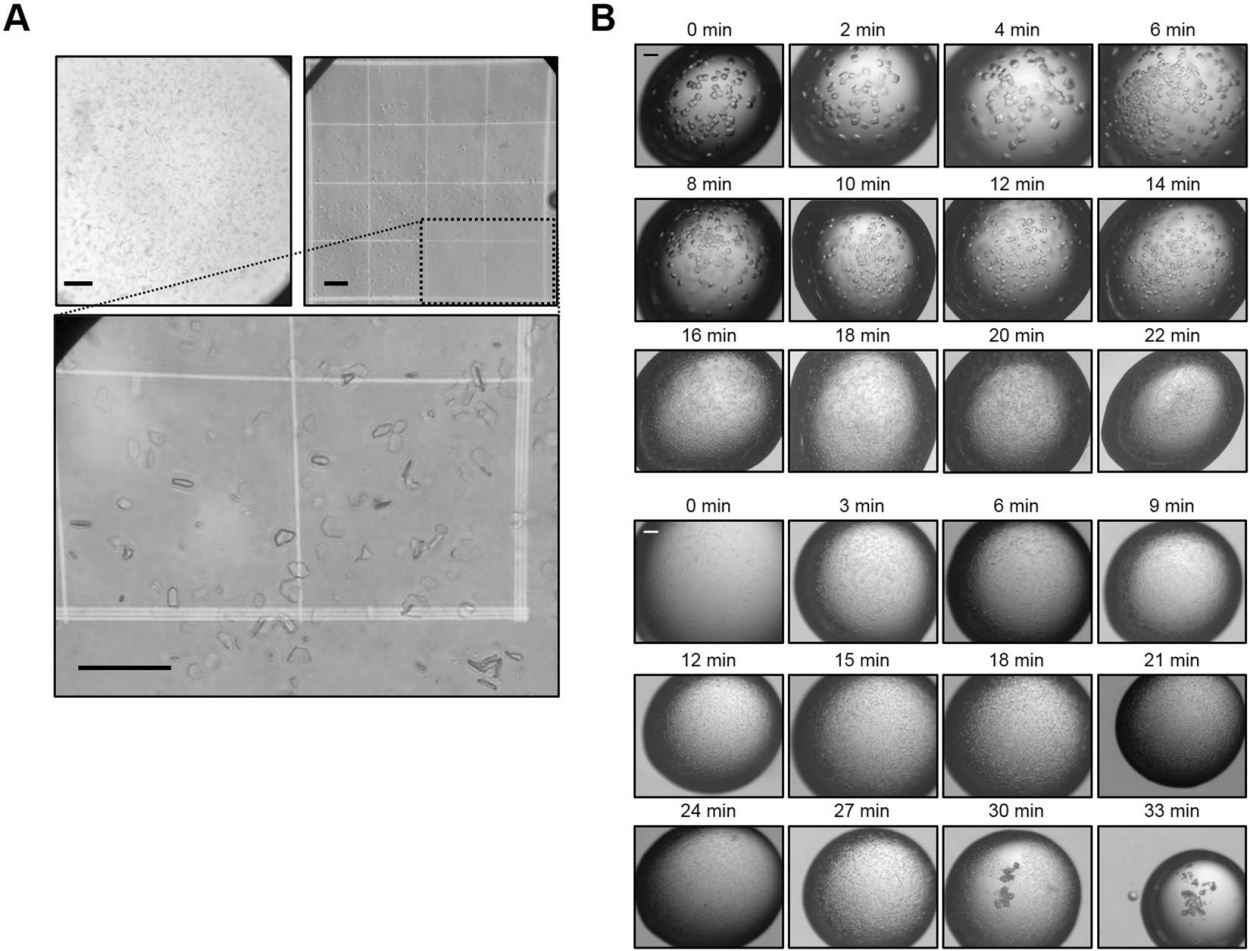
Microcrystallization. (**A**) HA microcrystals observed in a vapor diffusion drop by light microscope (upper-left panel), and transferred to hemocytometer (upper-right panel). The Neubauer chamber on the microscope stage of the hemocytometer showed a square ruled into 9 small squares, which were further divided into 16 smaller squares having sides of length 200 and 250 μm. Grid images in the dotted box are magnified in the lower panel. Scale bars: 100 μm. (**B**) Images of lysozyme (upper panel) and ferritin (lower panel) microcrystals in hanging drops obtained by supersaturation-controlled microcrystallization. Microcrystals were obtained at the highest density between 16 and 20 min for lysozyme and between 15 and 21 min for ferritin.

Purified recombinant HA protein was crystallized in 100 mM Tris-HCl (pH 8.0), 30% PEG 400, and 200 mM MgCl_2_ in 2 days, resulting in occasional formation of granular aggregates, frequent growth to form macrocrystals and the production of showers of tiny micron-sized crystals (Fig. S1, upper panel). To assess the size of nano- and microcrystals that are not clearly visible by light microscopy, a hemocytometer was used to show variable-sized microcrystals ranging in size from approximately 10 to 30 μm (Fig. 1A). However, microcrystallization of the HA protein was not reproducible, particularly in large-volume drops, and yielded microcrystals with different sizes. To improve the reproducibility of crystallization initially, rapid evaporation was applied to hanging drops; a crystallization solution containing a protein solution was dispensed on coverslips to produce hanging or sitting drops in a 24-well or 96-well plate. It was air-dried until white precipitates start to appear on the first coverslip, at which time the remaining coverslips were flipped over to seal the wells. Rapid evaporation produced microcrystals more reproducibly (Fig. S1, lower panel), with the yield reaching about 50%, compared to typical yields of less than 5%, based on the area occupied by microcrystals on the hemocytometer.

### Supersaturation-controlled microcrystallization

This rapid evaporation was further developed to test several microcrystallization conditions, where serial evaporation was controlled with a time delay (e.g., 2 min intervals) at the onset of microcrystallization. Lysozyme from chicken egg white was prepared at 55–75 mg/ml and the single crystallization condition of 100 mM sodium acetate (pH 4.8), 18% NaCl and 6–10% PEG 400 was used. Purified recombinant ferritin from *Escherichia coli* was prepared at 10 mg/mL and the single crystallization condition of 100 mM sodium acetate (pH 5.0) and 1 M NaCl with an additive buffer of 100 mM Tris-HCl (pH 8.0), 30% PEG 400, and 200 mM MgCl_2_ was used^23^. The protein and precipitant solutions were dispensed on coverslips in a drop size of 1.8 μL in a 24-well plate, air-dried to induce rapid evaporation of drops sequentially from 0 to 22 min at 2 min intervals for lysozyme (from 0 to 33 min at 3 min intervals for ferritin): the first drop containing lysozyme was air-dried for 2 min and sealed, the second one which had been dried for the same time was air-dried for additional 2 min and sealed, and the third one for additional 2 min and sealed, and so forth (Fig. S2). The effects of this time delay in the microcrystallization of the proteins were examined; microcrystals were usually obtained in 6–12 hrs at room temperature, and these typical microcrystals are shown in the figures. Strikingly, single large crystals of lysozyme appeared on the coverslips during the evaporation period of 0–14 min, whereas microcrystals were obtained at high density from 16 min to 20 min, when the lysozyme concentration was 55 mg/ml (Fig. 1B, upper panel). Smaller microcrystals of lysozyme at higher density were produced with higher concentrations of the lysozyme protein (55–75 mg/mL) (Fig. S3). As the concentration of lysozyme increased, the time delay required for microcrystallization also decreased. For ferritin, no single crystals were observed; instead, microcrystals with constant sizes were most typically obtained during the observed periods (Fig. 1B, lower panel). Supersaturation control was also found to produce a higher yield of relatively homogeneous microcrystals of ferritin, and microcrystals at the highest density were obtained at the evaporation period of 15–21 min. Then, supesaturation-controlled microcrystallization was tested for application to HA in sitting drops for the total duration of 11 min with a time delay of 30 sec. The highest yields of microcrystals of HA were observed at the duration of 14–15 min (Fig. S4). Microcrystals of ferritin and HA were again obtained in 6–12 hrs at room temperature. Taken together, these results strongly suggest that the protein microcrystallization conditions can be optimized to produce high-density microcrystals readily and rapidly by the supersaturation-controlled method, particularly when the single crystallization conditions are known.

### Characterization of microcrystals

**Figure 2:**
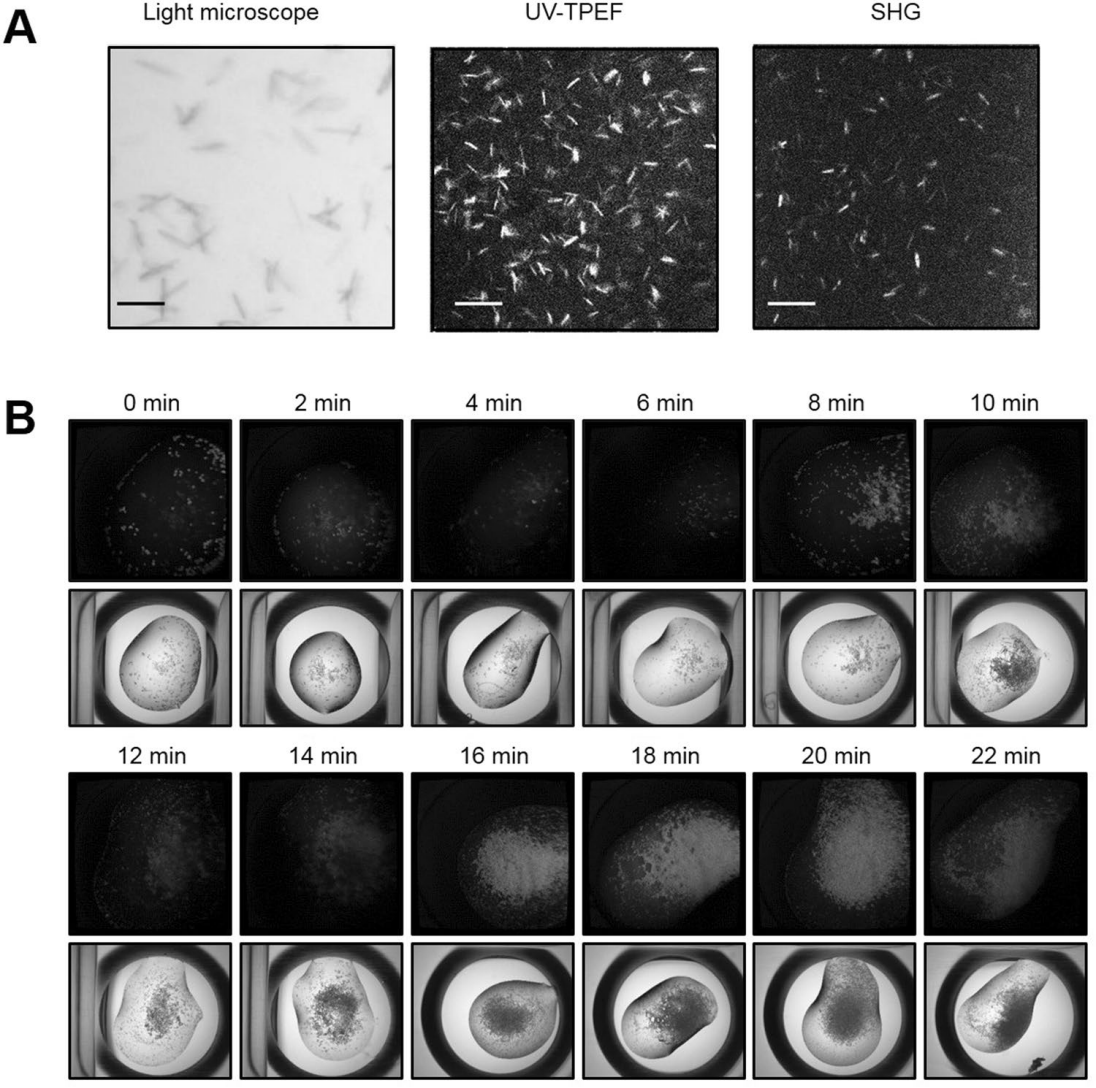
Characterization of microcrystals. (**A**) HA crystals detected by light microscopy, UV-TPEF, and SHG (left to right). Scale bars: 50 μm. (**B**) Lysozyme microcrystals detected by UV-TPEF and light microscopy in the crystallization buffer, 0.1 M sodium acetate (pH 4.8), 18% NaCl, and 6% PEG 400. Supersaturation-controlled microcrystallization was examined by using evaporation times of 0 to 22 min in 2 min delay steps.

To monitor the size, density, and quality of the obtained microcrystals, bright-field high-resolution microscopy, intrinsic UV fluorescence imaging, SONICC, X-ray powder diffraction, and TEM were used. The HA microcrystals showed positive ultraviolet two-photon excited fluorescence (UV-TPEF) and second-harmonic generation (SHG) signals. As the size of the HA microcrystals depended on the pH, those obtained at pH 7.0–7.25 were not visible in the SHG images, possibly due to their small sizes, in contrast to those obtained at pH 7.5–7.75 (Fig. 2A and Fig. S5). The lysozyme microcrystals also produced strong UV signals when they were monitored during the crystallization period (Fig. 2B). Microcrystals were clearly obtained at high density at the serial evaporation from 16 min to 20 min, consistent with the results from light microscopy. The ferritin microcrystals did not produce UV or SHG signals, possibly due to high symmetry packing (data not shown). The microcrystals of lysozyme and ferritin were shown to have sizes of 15–30 µm. As lysozyme and ferritin microcrystals are well known and monitored readily, the HA microcrystals obtained at pH 7.0–7.25 were further validated by powder diffraction. They produced powder diffraction rings to 6 Å (Fig. S6). Larger numbers of microcrystals produced higher resolution diffraction rings. Similar results were also reported in a recent powder diffraction study of lysozyme microcrystals^18^. Further, XFEL experiments were conducted to examine the diffraction quality of the microcrystals obtained from supersaturation-controlled microcrystallization. Representative images of HA and lysozyme microcrystals were found to diffract up to resolutions of 3.5 Å and 1.25 Å, respectively (Fig. S7). The poor quality of the HA diffraction data (low resolution limit) was mainly due to the sensitivity of HA microcrystals to physical mixing with agarose prior to loading into the LCP injector. Dissolved lysozyme microcrystals showed enzyme activity at pH 5.5, which was used for pH-dependent conformational change studies, and the refined structure also showed the tertiary structure very similar to that determined by synchrotron X-ray diffraction (Fig. S8).

### Visualization and analysis tools for microcrystals

**Figure 3:**
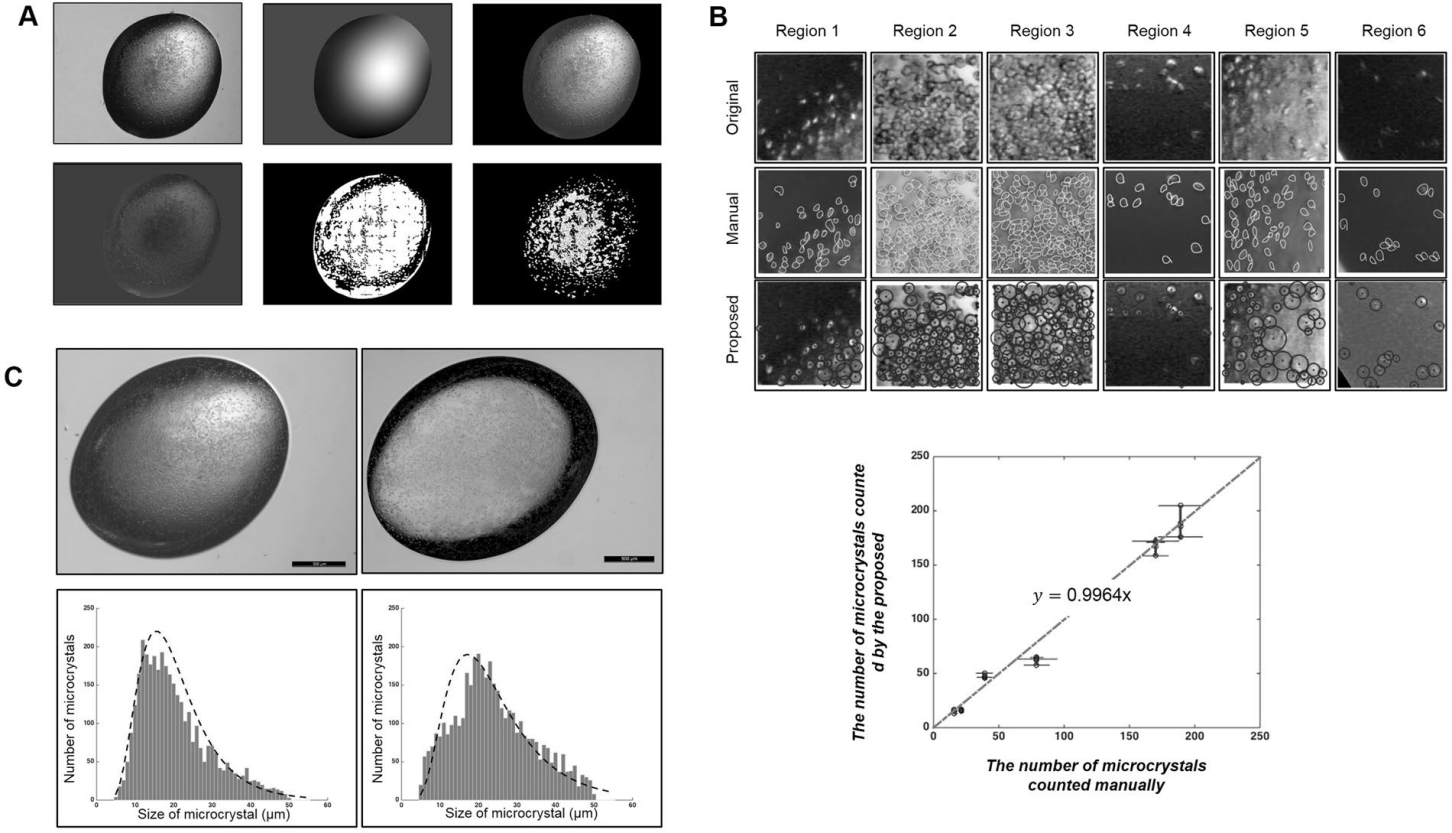
Images of protein microcrystallization drops. (**A**) Microcrystallization images processed by the proposed segmentation method: original drop image obtained by an ordinary light microscope, image bias map, and image after removal of the background (upper panel, left to right). The image after removal of the image bias and filling of the image background with the average of the edges of the bias corrected image, image after application of the localized fuzzy c-mean clustering algorithm, and segmented images were used to analyze the number of microcrystals (lower panel, left to right). The original image size was 2592 $\times$ 1944 pixels, and the localized fuzzy c-mean algorithm was applied to a small region of 200 $\times$ 200 pixels. (**B**) Comparison of manual and proposed microcrystal counting methods for selected regions of an image (upper panel): selected regions 1–6 of the original image (top row), manually marked microcrystals in each region (middle row), and microcrystals in each region marked by the proposed method (bottom row). The number of microcrystals counted manually was plotted against that of microcrystals counted by the proposed method (lower panel). The microcrystals were counted by three experts four times and by the proposed method three times. The data represent the means ± SD. (**C**) Original high-resolution (left) and ordinary light (right) microscopy images (upper panel) and the histogram analysis results of the high-resolution (left) and ordinary light (right) microscopy images (lower panel).

As shown in Fig. 2 and Figs S4–S5, the size, quality, and crystalline order of microcrystals can be monitored using bright-field high-resolution microscopy, UV fluorescence imaging, SONICC, and powder diffraction. Because the equipment for these measurements is not usually available in many ordinary laboratories, there is a genuine need to develop visualization tools to estimate the crystal size and density distributions. With progress in supersaturation-controlled microcrystallization, we developed a visualization tool to process the drop images by light microscopy; the original drop image had a spatially varying nonuniform bias near the boundary, from which a bias map was created by removing microcrystals and aggregates of proteins (Fig. 3A, top left and middle panels, respectively). The image size was 2592 $\times$ 1944 pixels in this case, and the image background was subtracted (Fig. 3A, top right). The image then showed a strong position-dependent bias in the drop region, which was also removed and filled with a background consisting of the average of the edges of the bias-corrected image (Fig. 3A, bottom left). The proposed method (the localized fuzzy c-mean clustering algorithm) was applied to segment the drop, generating approximately 20 divisions, each consisting of a small square region 200 $\times$ 200 pixels in size (Fig. 3A, bottom middle). These segmented images were used to analyze the number of microcrystals (Fig. 3A, bottom right). The numbers of microcrystals counted by the proposed method in six randomly selected regions were found to be very close to those counted manually (Fig. 3B, upper panel). For instance, the mean numbers of microcrystals counted manually in regions 1 to 6 in one image were 46.8, 167.4, 188.9, 16.2, 63.2, and 15.3 with standard deviations of 5.3, 12.3, 16.8, 1.8, 11.0, and 2.5 and those obtained by the proposed method, which was observer-independent and counted them automatically, were 39.0, 170.0, 189.0, 21.0, 79.0, and 16.0, respectively. For validation, three experts were selected to manually count the microcrystals four times, and the results were compared with those of the proposed method (Table S1). In addition, the proposed method can be used to monitor the size of crystals during supersaturation-controlled microcrystallization (Figs S9 and S10). The size distribution as a function of the evaporation time delay could reveal heterogeneous or homogeneous crystal sizes in different crystallization conditions of proteins, which is critical for optimization of microcrystal growth.

**Figure 4:**
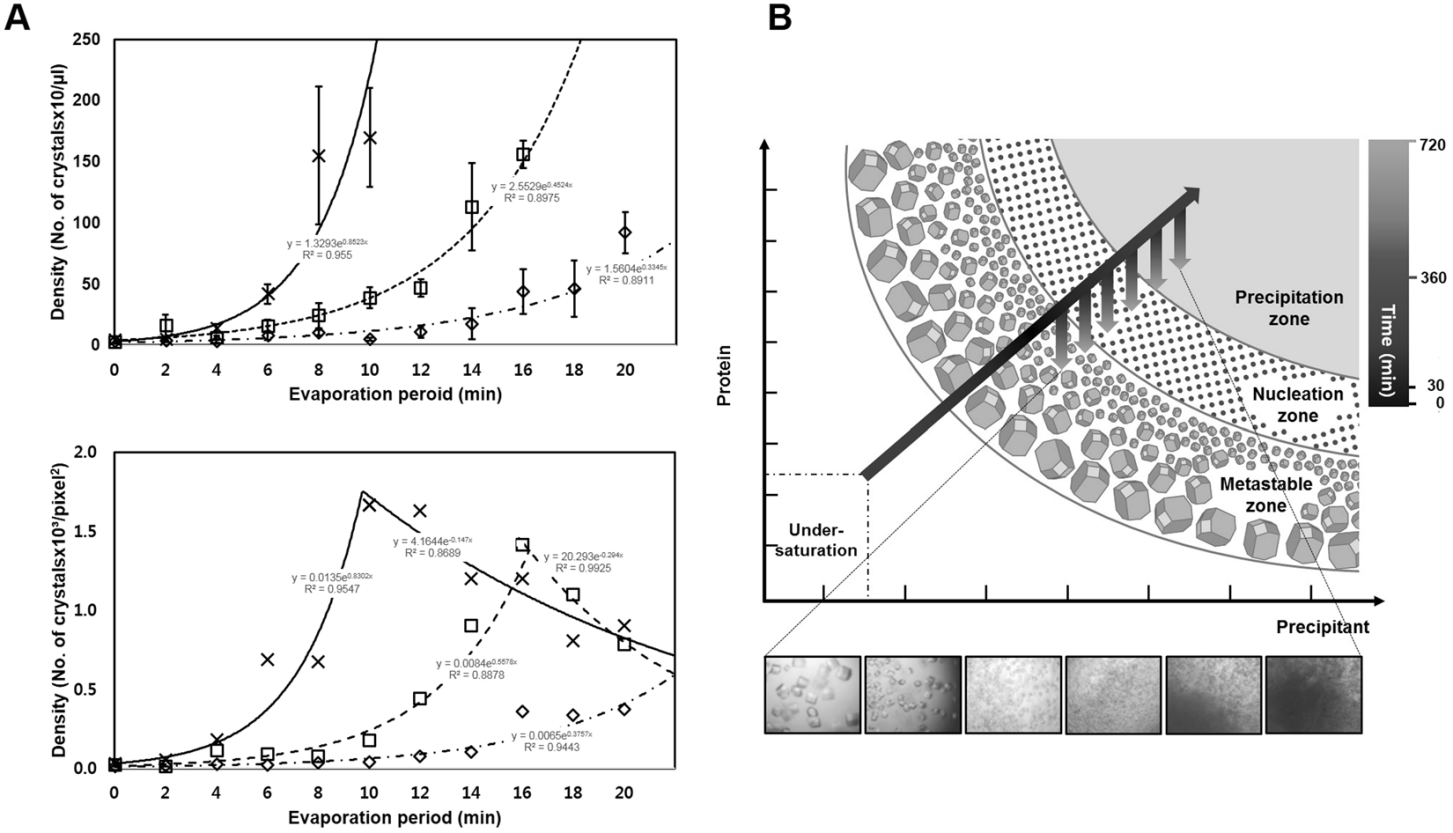
Supersaturation-controlled microcrystallization as a function of evaporation period for microcrystallization of lysozyme. (**A**) Plots of microcrystal density as a function of evaporation period for microcrystallization of lysozyme. The microcrystal densities were analyzed manually (upper panel) and by the proposed algorithm (lower panel). The lines are for a 1.8 μL drop with time periods from 0 to 22 min. Lysozyme protein concentrations of 55, 65 and 75 mg/mL were used, as shown by dashed dotted, dashed and continuous lines, respectively. The microcrystal densities at each time were calculated manually four times (upper panel) and by the proposed method three times (lower panel). The data represent the means ± SD. (**B**) Schematic representation of a two-dimensional phase diagram, illustrating estimated paths of microcrystallization, indicated by arrows, mediated by the supersaturation-controlled microcrystallization method. Long diagonal and short vertical arrows represent the changes in concentration for each time delay, resulting in the formation of single crystals, micro- or nanocrystals, and precipitates. The gray-scale in the arrows represents a real-time estimate from 0 to 720 min. The paths lead through different levels of supersaturation to rapidly reach the border between the metastable, nucleation, and precipitation zones.

To quantitatively evaluate the performance of the proposed counting method, first, the manual microcrystal counts were plotted against those obtained by the proposed method (Fig. 3B, lower panel). The slope of the line is 0.9964, and the correlation coefficient between the results of the manual and proposed methods was 0.9866. Second, we compared the proposed results with those obtained using high-resolution microscopy images (Fig. 3C). The nonuniformity was more pronounced in the high-resolution microscopy image than in the ordinary light microscopy. Nevertheless, histogram analysis showed that the mean sizes of microcrystals in the high- and low-resolution microscopy images were 22.5 and 25.6 μm with standard deviations of 0.8 and 2.5 μm, respectively, which were very similar. Particularly, the number of microcrystals in the high-resolution and ordinary light microscopy images were 4,086 and 3,556, respectively. Third, when the number of microcrystals was quantified as a function of the evaporation period at the onset of microcrystallization, the microcrystal counts by the manual and proposed methods showed consistent results, with tendency to increase at higher protein concentrations of lysozyme (Fig. 4A). As the evaporation period increased, the microcrystal densities of lysozyme and ferritin increased, albeit relatively slower increase in ferritin than lysozyme (Fig. S11). Notably, the proposed method could show that as the evaporation period increased further, the density of microcrystals started to decrease, when proteins are likely to form amorphous aggregates. The results suggest that the proposed method can distinguish between amorphous aggregates and crystalline protein samples, which is not easy for the manual task. Taken together, our proposed visualization and analysis methods provided an observer-independent tool to analyze the number and size of microcrystals automatically and accurately using light microscopy images. The proposed segmentation method was implemented using an Intel Xeon CPU E5-2690 v2 (3 GHz) with 128 GB of memory.

## Discussion

The emergence of XFEL-based SFX using the diffraction-before-destruction principle^7^ requires a novel paradigm for nano- or microcrystal screening of proteins and characterization of protein microcrystals. The micro-batch, free interface diffusion, and vapor diffusion methods are used for microcrystallization^10–14^. In this study, we developed supersaturation-controlled microcrystallization in a vapor diffusion mode to improve the reproducibility and screening efficiency in micro- or nanocrystallization. Hanging or sitting drops of aqueous microcrystallization solutions were evaporated with a precisely controlled time delay. Each well was sealed sequentially by coverslips containing drops to initiate microcrystallization. The number of nuclei in a crystallization trial is often limited to obtain single crystals by adding a protein-free buffer to hanging drops or by transferring hanging drops to new vapor diffusion media with lower concentrations of precipitating agent^24^. Microbatching under oil is also used to control the evaporation rate in protein crystallization, in which evaporation is arrested in the drops under oil^25,26^. As such, slowing the rate of supersaturation results in large single crystals, which has typically been used for synchrotron studies. In contrast, our supersaturation-controlled microcrystallization approach increased the level of supersaturation rapidly at the onset of crystallization, which is opposite in direction to conventional methods of producing single crystals. Nucleation in protein crystallization depends on the level of supersaturation, which is significantly decreased in nanoliter volumes^27^, particularly if nucleation is homogeneous^28^. In this respect, it was remarkable that evaporation-induced supersaturation could compensate for the decreasing chance of nucleation in a nano- or micro-volume drops and enable the point of nucleation to be reached rapidly. The level of supersaturation at which micro- or nanocrystals were obtained was indeed very successfully reached by controlled nucleation. Furthermore, difficult-to-obtain protein crystals are often not reproducible under the same crystallization conditions, particularly in microcrystallization in large volumes. This supersaturation-controlled microcrystallization method enhanced the yield and rate of crystallization significantly, producing microcrystals at high density in 6–12 hrs. When the initial crystallization conditions for single crystals are known, this method is particularly useful for optimizing the micro- or nanocrystallization conditions.

Crystal nucleation normally reduces the local protein concentrations in drops, whereas supersaturation-controlled microcrystallization is not likely to reduce the protein concentrations significantly, because serial evaporation occurs with a time delay. Nucleation is thus likely to occur continuously, resulting in massive nucleation. We propose, as shown in a crystal phase diagram (Fig. 4B), that supersaturation-controlled microcrystallization allows protein solutions in drops to be evaporated serially with a time delay, so that they initially cross from the metastable zone to the nucleation zone, inducing nucleation. Delayed evaporation of drops further brings the protein solution closer to the border between nucleation and precipitation zones, so continuous nucleation can be maintained. The microcrystal density clearly increased as the delayed evaporation increased, and this effect was more pronounced as the protein concentration increased. Importantly, the exact time at which nucleation or precipitation occurs can be determined readily by monitoring the drops; the conditions longer than the maximum time delay result in the formation of protein precipitates in drops, whereas those at early time delay results in clear solution or single crystals. Interestingly, a rapid nucleation is reached in a short time frame, whereas further nucleation is taking place in a longer time frame. It is noteworthy that this method offers a significantly higher nucleation rate and yield of microcrystals of HA, ferritin, and lysozyme. When it was applied under the well-known single crystallization condition of lysozyme, single large crystals appeared early during the evaporation period, and were replaced by microcrystals at higher density later. Therefore, this method can be used to estimate the extent of the nucleation zone in the diagram, depending on the evaporation period to control supersaturation, and, more importantly, it can be performed in practical time frames to initiate micro- or nanocrystallization of various proteins.

Characterization of protein microcrystals by size, density, and homogeneity requires efficient methods such as DLS, SHG, powder diffraction, and TEM^15–19^. SONICC in combination with UV-TPEF is a powerful method of obtaining positive signals from HA and lysozyme microcrystals. However, the smaller lysozyme microcrystals showed little or no signal, and no signals were detected from ferritin microcrystals with high symmetry. Powder diffraction patterns with a small spacing, indicating a large protein molecule, have recently provided evidence of how well microcrystals are ordered and how suitable they are for SFX experiments at XFELs^18^. Nevertheless, reliable assessment of microcrystal size and density using the above methods is not readily available in many ordinary laboratories. The visualization tools presented in this study adopt the localized fuzzy c-mean clustering and bias correction algorithms to segment light microscopy images of crystallization in hanging or sitting drops^29–31^. It was known that each particle in the image exhibited a consistent increase in pixel intensity in relation to its background. The number of microcrystals based on the segmented images was very close to that obtained by manual counting, with a correlation coefficient of 0.9866. Furthermore, our comparative analysis of the results of the proposed method and high-resolution microscopy images indicated that the numbers and mean sizes of the microcrystals were very similar, suggesting the strength of our proposed method based solely on light microscopy images.

In conclusion, our supersaturation-controlled microcrystallization method shows promise for optimizing the nucleation conditions of microcrystals, once the crystallization conditions are known, and our visualization and analysis tools can characterize the size and density distributions of nano- or microcrystals using ordinary light microscopy images. Our supersaturation-controlled microcrystallization and visualization and analysis tools will provide universal access to successful XFEL studies.

## Materials and Methods

### Cloning and baculovirus production

HA protein was produced in insect cells using recombinant baculovirus expression vectors. The isolated DNA of HA derived from a seasonal strain, A/Thailand/CU44/2006 (H1N1), was amplified using polymerase chain reaction (PCR).

### Protein expression and purification

A baculovirus containing the CU44 wild type HA gene was used to infect *Trichoplusia ni* High Five cells. The culture medium was harvested and applied to a nickel-nitrilotriacetic acid (Ni-NTA) affinity column. The eluted precursor HA protein was dialyzed and hydrolyzed by thrombin to remove the foldon region and 6xHis tag. The active form of HA was purified by Mono Q ion-exchange chromatography and Superdex 200HR size exclusion chromatography, which was concentrated to 15–25 mg/mL for crystallization. The wild-type ferritin gene of *E. coli* strain K12 and its mutant S20A were prepared as described previously^23^. Protein expression was induced by addition of isopropyl β-D-1-thiogalactopyranoside and the supernatant was purified by Ni-NTA affinity chromatography and gel filtration chromatography. The purified HA and ferritin and purchased lysozyme proteins were analysed by sodium dodecyl sulfate-polyacrylamide gel electrophoresis (SDS-PAGE).

### Microcrystallization of proteins

The purified recombinant HA (15 mg/mL) was screened by the hanging or sitting drop vapor diffusion method. HA (0.3–1 µL) was mixed with 0.3 µL of screening solution at 100 mM Tris-HCl (pH 8.0), 30% PEG 400, 200 mM MgCl_2_ produced showers of tiny crystals. *E. coli* ferritin was prepared in a 50 mM HEPES-HCl, pH 6.5 and 200 mM NaCl buffer at 10 mg/mL and mixed at a ratio of 3:2:1 (v/v/v) with a precipitant solution consisting of 100 mM sodium acetate trihydrate, pH 5.0, and 1 M NaCl and an additive buffer consisting of 100 mM Tris-HCl, pH 8.0, 30% PEG 400, and 200 mM MgCl_2_. Lysozyme in 100 mM sodium acetate pH 4.8 at 55, 65 or 75 mg/mL was mixed with a precipitation buffer consisting of 100 mM sodium acetate, pH 4.8, 18% NaCl and 6% PEG 400 at a ratio of 1:3.

### Supersaturation-controlled microcrystallization

For lysozyme, evaporation periods from 0 to 22 min with a time delay of 2 min were used, and microcrystals at high density were obtained between 16 and 20 min. Microcrystals could be obtained at high density at the same time by evaporation with higher concentrations of proteins. When supersaturation-controlled crystallization was used, lysozyme microcrystals showed decreased sizes and increased crystal density with increased evaporation time. In ferritin, evaporation proceeded sequentially from 0 to 33 min with a time delay of 3-min after hanging drops were produced on cover slips. The sizes of microcrystals, which were obtained at high density, were almost constant. HA was microcrystallized in sitting drops for the periods of 0–15 min with a time delay of 30 sec. The highest yield of microcrystals of HA was observed at 14–15 min

### Hemocytometry and high-resolution microscopy

To assess the size of nano- and microcrystals that are not clearly visible by light microscopy, the crystal samples were taken directly from the crystal plate to the hemocytometer. The hemocytometer image showed HA microcrystals ranging in size from approximately 10 to 30 μm. Microcrystals of HA, ferritin and lysozyme were also observed by a Nikon SMZ800 (Nikon, Tokyo, Japan) or LEICA M205 A (Leica, Wetzlar, Germany) to generate high-resolution microcrystal images.

### SHG imaging, TEM and powder diffraction of protein microcrystals

The crystallinity of microcrystals of HA, ferritin and lysozyme was assessed by SHG and UV-TPEF imaging using a SONICC imager. TEM images of microcrystals were observed with a Hitachi H7600 transmission electron microscope, and powder diffraction data were obtained at BL38B1 at SPring8 (Hyogo, Japan) or at BL-1A at the Photon Factory (Tsukuba, Japan).

### XFEL data collection and analyses

SFX experiments were performed at experimental stations CXI^32,33^ at the Linac Coherent Light Source (LCLS)^34^ at SLAC National Accelerator Laboratory and at the PAL-XFEL^35^. The LCLS and PAL-XFEL were operated at wavelengths of 1.305 Å (9.5 keV) and 1.26 Å (9.78 keV), and single-shot diffraction patterns of randomly oriented crystals were recorded at 120 Hz with the Cornell–SLAC Pixel Array Detector positioned 160 mm from the sample at the LCLS and at 10 Hz with a Rayonix MX225-HS 87.5 mm from the sample at the PAL-XFEL.

For HA, a total of 662,438 images were collected in 5.6 hrs, of which 10,270 were identified as crystal diffraction patterns by Cheetah^36^, for an average hit rate of 1.6%. Autoindexing and structure-factor integration of the crystal hits were performed using CrystFEL^37^. For lysozyme, a total of 418,719 images were collected in 20 hrs, of which 107,754 were identified as crystal diffraction patterns by NanoPeakCell^38^, for an average hit rate of 25.7%. Autoindexing and structure-factor integration of the crystal hits were performed using CrystFEL^37^.

### Determination of structure and enzyme activity

The initial model of lysozyme was built using molecular replacement and AutoBuild from the PHENIX suite^39^, employing a previously solved structure (PDB 4Z98) as a search model. Lysozyme model building was performed in the Coot program^40^. The *R* and *R*_free_ values for the final structure were 19.3% and 23.1%, respectively, using the PHENIX program^39^. Ramachandran analysis revealed 99.2%, 0.8% and 0% in the favored, allowed, and outlier regions, respectively. The structural figures were generated with PyMOL (http://www.pymol.org/). The data quality and refinement statistics are presented in Table 1.

**Table 1:** Data collection and refinement statistics.

Lysozyme	
**Data statistics**	
Space group	P4_3_2_1_2
**Cell dimensions**	
*a*, *b*, *c* (Å)	79.0, 79.0, 38.0
α, β, γ (°)	90.0, 90.0, 90.0
Resolution (Å)^*^	27.9–1.9 (2.09–1.9)
Number of reflections	5,070,957
Number of unique miller indices	18,131
Multiplicity^*^	279.25 (189.6)
R_split_ (%)^*†^	19.3 (32.9)
CC_½_ (%)^*^	0.92 (0.85)
<I/σ(I)>^*^	6.69 (2.87)
**Refinement statistics**	
Resolution (Å)	1.9
R_work_/R_free_ (%)	19.3/23.1
**Ramachandran (%)**	
Favored	99.2
Allowed	0.8
Disallowed	0
**R.m.s. deviations from ideal**	
Bond lengths (Å)	0.004
Bond angles (°)	0.634
Number of Waters	42

^*^Values in parentheses are for the highest-resolution shell.

^†^Rsplit = $$1/\surd 2\frac{{\Sigma }_{{hkl}}|{I}_{{even}}-{I}_{{odd}}|}{1/2{\Sigma }_{{hkl}}|{I}_{{even}}+{I}_{{odd}}|}$$.

Lysozyme activity resulting in the lysis of the *Micrococcus lysodeikticus* cells was monitored by the absorbance change at 450 nm in 50 mM sodium acetate (pH 5.5), using the Sigma-Aldrich lysozyme detection kit (Catalog Number LY0100)

### Visualization and analysis tools for microcrystals

The segmentation counting method implemented in MATLAB 2014b (Natick, MA, USA) consisted of two major steps; image uniformity correction and image segmentation. Briefly, the non-uniformity image bias correction algorithm was applied as follows: First, the original images were smoothened, the image background was removed using Otsu’s threshold method^30^, and non-uniform image bias maps were calculated by applying morphological opening to the original images^31^. Next, the image bias was subtracted to correct the non-uniformity, yielding the bias-corrected image, and then the image background was filled with the average of the edges of the bias-corrected image.

The localized fuzzy c-mean clustering algorithm was applied for image segmentation. The fuzzy c-mean clustering algorithm, which generates fuzzy partitions and prototypes for numerical data, was useful for corroborating known substructures or suggesting substructures in the original data^29^. A localized fuzzy c-mean algorithm was used to process fuzzy c-mean clustering in a small region, which was then divided from the image after the algorithm for correcting the non-uniformity bias was applied in the drop region of the image. This algorithm could avoid nonstationary images and provide good segmentation even when the regions of the image to be segmented had a wide range of intensities.

## Electronic supplementary material

Supplementary information
