# Author Correction: Supersaturation-controlled microcrystallization and visualization analysis for serial femtosecond crystallography

**DOI:** 10.1038/s41598-018-24178-5

**Published:** 2018-04-17

**Authors:** Dan Bi Lee, Jong-Min Kim, Jong Hyeon Seok, Ji-Hye Lee, Jae Deok Jo, Ji Young Mun, Chelsie Conrad, Jesse Coe, Garrett Nelson, Brenda Hogue, Thomas A. White, Nadia Zatsepin, Uwe Weierstall, Anton Barty, Henry Chapman, Petra Fromme, John Spence, Mi Sook Chung, Chang-Hyun Oh, Kyung Hyun Kim

**Affiliations:** 1 Department of Biotechnology & Bioinformatics, Korea University, Sejong, Korea; 2 Department of Electronics & Information Engineering, Korea University, Sejong, Korea; 3 Department of Structure and Function of Neural Network, Korea Brain Research Institute, Daegu, Korea; 4 Department of Chemistry, Arizona State University, Tempe, Arizona USA; 5 Department of Physics, Arizona State University, Tempe, Arizona USA; 6 Biodesign Center for Applied Structural Discovery, Arizona State University, Tempe, Arizona USA; 7 Center for Free-Electron Laser Science, Deutsches Elektronen-Synchrotron DESY, Hamburg, Germany; 8 Department of Food and Nutrition, Duksung Women’s University, Seoul, Korea

Correction to: *Scientific Reports* 10.1038/s41598-018-20899-9, published online 07 February 2018

The original version of this Article contained a typographical error in the spelling of the author Garrett Nelson, which was incorrectly given as Gerrett Nelson. This has now been corrected in the PDF and HTML versions of the Article, and in the accompanying Supplementary Information file.

