# Supplementary material for: Supersaturation-controlled microcrystallization and visualization analysis for serial femtosecond crystallography
Source: Sci Rep. 2018 Feb 7;8:2541. doi: 10.1038/s41598-018-20899-9 (PMC5803221; doi:10.1038/s41598-018-20899-9)
Supplement: Supplementary file 1 — Supplementary information [file 41598_2018_20899_MOESM1_ESM.docx]

Supplementary Materials

Supersaturation-controlled microcrystallization and visualization analysis for serial femtosecond crystallography

**Dan Bi Lee^1,*^, Jong-Min Kim^2,*^, Jong Hyeon Seok^1^, Ji-Hye Lee^1^, Jae Deok Jo^1^, Ji Young Mun^3^, Chelsie Conrad^4^, Jesse Coe^4^, Garrett Nelson^5^, Brenda Hogue**^6^**, Thomas A. White^7^, Nadia Zatsepin^5^, Uwe Weierstall^5^, Anton Barty^7^, Henry Chapman^7^, Petra Fromme**^4^**, John Spence^5^, Mi Sook Chung^8^, Chang-Hyun Oh^2,**^, and Kyung Hyun Kim^1,**^**

*^1^Department of Biotechnology & Bioinformatics, ^2^Department of Electronics & Information Engineering, Korea University, Sejong, Korea, ^3^Department of Biomedical Laboratory Science, College of Health Sciences, Eulji University, Gyeonggi-Do, Korea, ^4^Department of Chemistry, ^5^Department of Physics, ^6^Biodesign Center for Applied Structural Discovery, Arizona State University, Arizona, U.S.A., ^7^Center for Free-Electron Laser Science, Deutsches Elektronen-Synchrotron DESY, Hamburg, Germany, ^6^Department of Food and Nutrition, Duksung Women’s University, Seoul, Korea.*

**Supplementary information**

Contents

Materials and methods

References

Table S1

Figures S1-S11

Materials and Methods

**Cloning and baculovirus production**

HA protein was produced in insect cells using recombinant baculovirus expression vectors. The isolated DNA of HA derived from a seasonal strain, A/Thailand/CU44/2006 (H1N1), was amplified using polymerase chain reaction (PCR). An HA gene (1-503 of HA0) was cloned in pFastBac^TM^HT A (Invitrogen, Carlsbad, CA, USA) downstream of the gp67 secretion signal sequence of the transfer vector pAcGP67A (BD Biosciences, MA, USA). The plasmid encoding the HA gene was amplified in *E. coli* strain DH5α and the recombinant bacmid was generated according to the Bac-to-Bac expression system protocol (Invitrogen, CA, USA). The sequence was confirmed by automated sequencing (Macrogen, Seoul, Korea). *Spodoptera frugiperda* (Sf-9) insect cells (Invitrogen, CA, USA) were transfected with the recombinant bacmid using Cellfectin II (Invitrogen, CA, USA). The transfected cells were harvested at 96 hrs post-transfection and centrifuged at 2,000 rpm for 15 min to obtain the baculovirus expressing the recombinant HA in the supernatant. The transfection efficiency was confirmed by PCR after extraction of DNA from 400 μL of virus (cell supernatant).

**Protein expression and purification**

A baculovirus containing the CU44 wild type HA gene was used to infect suspension cultures of *Trichoplusia ni* High Five cells. After 3-4 days at 28°C, the culture medium was harvested and applied to a nickel-nitrilotriacetic acid (Ni-NTA) affinity column equilibrated with a working buffer (20 mM Tris-HCl, pH 8.0, and 100 mM NaCl). The precursor HA protein was eluted after the column was washed with a buffer containing 30 mM imidazole, which was dialyzed against 20 mM Tris-HCl (pH 8.0) and 20 mM NaCl, and hydrolyzed by thrombin to remove the foldon region and 6xHis tag with the addition of 1 μg/mL L-tosylamide-2-phenylethyl chloromethyl ketone-treated trypsin (Sigma-Aldrich, St. Louis, MO, USA) for cleavage of HA0 into HA1 and HA2 for 4-5 h at 4°C. The active form of HA was purified by Mono Q ion-exchange chromatography and Superdex 200HR size exclusion chromatography in 50 mM Tris-HCl (pH 8.0) and 100 mM NaCl, in instruments connected to an ÄKTA FPLC system (GE HealthCare, Milwalkee, USA). The HA was concentrated in an Amicon 10,000 MWCO concentrator (Merck Millipore, Billerica, MA, USA) to 15-25 mg/mL for crystallization.

The wild-type ferritin gene of *E. coli* strain K12 (Korean Culture Center of Microorganisms, Seoul, Korea) and its mutant S20A were prepared as described previously^1^. Protein expression was induced by addition of isopropyl β-D-1-thiogalactopyranoside to a final concentration of 0.4 mM, and the supernatant was purified by Ni-NTA affinity chromatography and gel filtration chromatography using an ÄKTA system with a Superdex 200 Hiload 16/60 column (GE Healthcare, Uppsala, Sweden) in 20 mM Tris-HCl (pH 8.0). The purified HA and ferritin and purchased lysozyme proteins were denatured by heating at 90°C for 10 min in the sample buffer and separated by 12.5% sodium dodecyl sulfate-polyacrylamide gel electrophoresis (SDS-PAGE) for 40 min. The electrophoresis was performed in 25 mM Tris-HCl buffer (pH 8.8). After the gels were run, they were stained in a staining solution with 1% Coomassie Blue R-250 for 1 hr at room temperature, and gently agitated in destaining solution until the background became clear.

**Microcrystallization of proteins**

The purified recombinant HA (15 mg/mL) was screened by the hanging or sitting drop vapor diffusion method using commercial kits (Hampton Research, Aliso Viejo, CA, USA). HA (0.3-1 µL) was mixed with 0.3 µL of screening solution at 24°C. One of the crystallization conditions, 100 mM Tris-HCl (pH 8.0), 30% PEG 400, 200 mM MgCl_2_, and 25 mg/mL of HA, produced showers of tiny crystals, and another condition, 100 mM Tris-HCl (pH 7.5), 20% PEG 2000 and 20 mg/mL of HA, produced single crystals, as large as >200 μm. Microcrystals grown in PEG400 had different sizes depending on the solution pH between 7.0 and 8.0 using 1 M Tris-HCl.

*E. coli* ferritin was prepared in a 50 mM HEPES-HCl, pH 6.5 and 200 mM NaCl buffer at 10 mg/mL and mixed at a ratio of 3:2:1 (v/v/v) with a precipitant solution consisting of 100 mM sodium acetate trihydrate, pH 5.0, and 1 M NaCl and an additive buffer consisting of 100 mM Tris-HCl, pH 8.0, 30% PEG 400, and 200 mM MgCl_2_.

Lysozyme (L6876, Sigma-Aldrich, St.Louis, MO, USA) was dissolved in a buffer of 100 mM sodium acetate pH 4.8 at 55, 65 and 75 mg/mL, and then mixed with a precipitation buffer consisting of 100 mM sodium acetate, pH 4.8, 18% NaCl and 6% PEG 400 at a ratio of 1:3. After mixing, the hanging drop with a drop size of 1.8 μL of ferritin and lysozyme was divided on a cover slip in a 24-well plate each.

**Supersaturation-controlled microcrystallization**

To improve the reproducibility of HA microcrystallization, a supersaturation-controlled microcrystallization protocol was designed as follows. A crystallization setup of 1:1 protein:precipitant solutions by volume in 100 mM Tris-HCl (pH 8.0), 30% PEG 400, and 200 mM MgCl_2_ was maintained on coverslips in a 24-well plate and air-dried sequentially with a time delay, depending on the protein; the coverslips were then flipped over, and each well was sealed.

For lysozyme, evaporation periods from 0 to 22 min with a time delay of 2 min were used, and microcrystals at high density were obtained between 16 and 20 min. Microcrystals could be obtained at high density at the same time by evaporation with higher concentrations of proteins. When supersaturation-controlled crystallization was used, lysozyme microcrystals showed decreased sizes and increased crystal density with increased evaporation time. In ferritin, evaporation proceeded sequentially from 0 to 33 min with a time delay of 3-min after hanging drops were produced on cover slips. The sizes of microcrystals, which were obtained at high density, were almost constant. HA was microcrystallized in sitting drops for the periods of 0-11 min with a time delay of 30 sec. The highest yield of microcrystals of HA was observed at 14-15 min

**Hemocytometry and high-resolution microscopy**

To assess the size of nano- and microcrystals that are not clearly visible by light microscopy, the crystal samples were taken directly from the crystal plate to a grid on a hemocytometer slide (Marienfeld-Superior, Lauda-Königshofen, Germany). The Neubauer chamber on the microscope stage showed a square ruled into 9 small squares, each of which was further divided into smaller squares having sides of length 200 and 250 μm. The hemocytometer image showed HA microcrystals ranging in size from approximately 10 to 30 μm. Microcrystals of HA, ferritin and lysozyme were also observed by a Nikon SMZ800 (Nikon, Tokyo, Japan) or Olympus SZX or CK2 microscope (Olympus, Shinjuku, Japan) or LEICA M205 A (Leica, Wetzlar, Germany) to generate high-resolution microcrystal images.

**SHG imaging of protein microcrystals**

The crystallinity of microcrystals of HA, ferritin and lysozyme was assessed by SHG and UV-TPEF imaging using a SONICC imager (Formulatrix, Bedford, MA, U.S.A.) at the Pohang Accelerator Laboratory (PAL)-XFEL (Kyungbook, Korea). These complementary methods allowed the detection of chiral crystals and protein crystals, respectively. SHG imaging often gave false negative results in protein crystals that were packed at high symmetry or had small sizes (under 15 µm). Microliter aliquots of HA microcrystals grown in vials in 100 mM Tris-HCl (pH 7.0, 7.25, 7.5, and 7.75), 30% PEG 400, and 200 mM MgCl_2_, lysozyme grown in 100 mM sodium acetate, pH 4.8, 18% NaCl, and 6% PEG 400, and ferritin grown in 100 mM sodium acetate trihydrate, pH 5.0, and 1 M NaCl were transferred onto the drop wells of a MRC2 plate. Signals were collected first at medium SHG power (350 mW laser power) and then at high SHG power (450 mW laser power) to detect the smallest possible crystals.

**Transmission electron microscopy (TEM) and powder diffraction**

The microcrystals were applied to freshly charged carbon-coated grids and quickly stained with a 2% uranyl acetate solution. The stained microcrystals were observed with a Hitachi H7600 transmission electron microscope (Hitachi, Tokyo, Japan) at 80 kV at a magnification of 700×. A 50 μL aliquot of crystal slurry was spun down at room temperature to pellet the crystals. The liquid surrounding the pellet was aspirated off and replaced with a cryosolution of 5% ethylene glycol, and the sample was re-centrifuged to pelle t the crystals^2^. Most of the supernatant was removed from the crystals. For powder diffraction, a large volume of the crystals was pipetted onto a MiTeGen mesh, plunged into liquid nitrogen, and placed on a standard crystallography mount at BL38B1 at SPring8 (Hyogo, Japan) or at BL-1A at the Photon Factory (Tsukuba, Japan). A beam with a wavelength of 1.000 Å and a spot size of 80 μm × 110 μm or 200 μm × 110 μm was used with a 20 sec exposure time, and powder diffraction data were collected at a crystal-to-detector distance of 400 or 500 mm. Two-dimensional diffraction images were recorded on an ADSC Quantum 315r CCD. All images were viewed using iMosflm^3^.

**XFEL data collection**

SFX experiments were performed at experimental stations CXI^4,5^ at the Linac Coherent Light Source (LCLS)^6^ at SLAC National Accelerator Laboratory and at the PAL-XFEL^7^. The LCLS and PAL-XFEL were operated at wavelengths of 1.305 Å (9.5 keV) and 1.26 Å (9.78 keV), delivering individual X-ray pulses of nominally 35 and 40 fs pulse duration, respectively. HA and lysozyme microcrystals were injected using LCP medium at flow rates of 170 and 135 nL/min into the beam focus region at the LCLS and PAL-XFEL inside vacuum and helium chambers, respectively. Single-shot diffraction patterns of randomly oriented crystals were recorded at 120 Hz with the Cornell–SLAC Pixel Array Detector positioned 160 mm from the sample at the LCLS and at 10 Hz with a Rayonix MX225-HS 87.5 mm from the sample at the PAL-XFEL.

For HA, a total of 662,438 images were collected in 5.6 hrs, of which 10,270 were identified as crystal diffraction patterns by Cheetah^8^, for an average hit rate of 1.6%. The peak-detection parameters and experimental geometry were optimized to ensure the best quality of peak finding and indexing. Autoindexing and structure-factor integration of the crystal hits were performed using CrystFEL^9^. For lysozyme, a total of 418,719 images were collected in 20 hrs, of which 107,754 were identified as crystal diffraction patterns by NanoPeakCell^10^, for an average hit rate of 25.7%. The peak-detection parameters and experimental geometry were optimized to ensure the best quality of peak finding and indexing. Autoindexing and structure-factor integration of the crystal hits were performed using CrystFEL^10^.

**Determination of structure and enzyme activity**

The initial model of lysozyme was built using molecular replacement and AutoBuild from the PHENIX suite^11^, employing a previously solved structure (PDB 4Z98) as a search model. Lysozyme was built in the Coot program^12^. The *R* and *R*_free_ values for lysozyme at room temperature were 19.3% and 23.1%, respectively, using the PHENIX program^11^. Ramachandran analysis revealed 99.2%, 0.8% and 0% in the favored, allowed, and outlier regions, respectively. The structural figures were generated with PyMOL (http://www.pymol.org/). The data quality and refinement statistics are presented in Table 1.

Lysozyme activity resulting in the lysis of the *Micrococcus lysodeikticus* cells was monitored by the absorbance change at 450 nm in 50 mM sodium acetate (pH 5.5), using the Sigma-Aldrich lysozyme detection kit (Catalog Number LY0100). Briefly, 800 µL of the *Micrococcus* cell suspension was incubated with 30 µL of lysozyme solution in reaction buffer was used in 50 mM sodium acetate (pH 5.5 ), and the decrease in A450 was recorded.

**Visualization and analysis tools for microcrystals**

The segmentation counting method was implemented in MATLAB 2014b (Natick, MA, USA). It consisted of two major steps; image uniformity correction and image segmentation. Correcting the non-uniform bias in the drop region of the image was an important step that extracted small but precise features of microcrystals with varying image bias. Briefly, the non-uniformity image bias correction algorithm was applied as follows: First, the original images were smoothened, the image background was removed using Otsu’s threshold method^13^, and non-uniform image bias maps were calculated by applying morphological opening to the original images^14^. Next, the image bias was subtracted to correct the non-uniformity, yielding the bias-corrected image, and then the image background was filled with the average of the edges of the bias-corrected image.

The localized fuzzy c-mean clustering algorithm was applied for image segmentation. The fuzzy c-mean clustering algorithm, which generates fuzzy partitions and prototypes for numerical data, was useful for corroborating known substructures or suggesting substructures in the original data^15^. The clustering criterion was used to aggregate subsets of data using a generalized least-square objective function. For application to microcrystal counting, however, the proposed algorithm was modified so that it could find the feature points of overlapping and small structures. A localized fuzzy c-mean algorithm was used to process fuzzy c-mean clustering in a small region, which was then divided from the image after the algorithm for correcting the non-uniformity bias was applied in the drop region of the image. The entire image segmentation procedures using the localized fuzzy c-mean clustering algorithm was as follows:^16^ a spatial adaptive filter is applied to the bias-corrected image to minimize the effect of noise,^17^ the image background is filled with the average value of the edges of the drop region in the bias-corrected image. In this step, the feature points cannot be detected in small regions in the localized fuzzy c-mean clustering algorithm scheme, which is composed of the image background and the drop region of the image.^18^ The localized fuzzy c-mean algorithm is applied, and^19^ the labeled image is refined by post-processing. The center of each region is allocated and if no center can be found, the region is discarded. Regions with a total number of points beyond a reasonable range of microcrystal sizes were also eliminated. This algorithm could avoid nonstationary images and provide good segmentation even when the regions of the image to be segmented had a wide range of intensities.

References

1.  Kim, S., et al. Structural basis of novel iron-uptake route and reaction intermediates in ferritins from Gram-negative bacteria. *J. Mol. Biol*. **428,** 5007-5018 (2016).

2. Stevenson, H. P., et al. Use of transmission electron microscopy to identify nanocrystals of challenging protein targets. *Proc. Natl. Acad. Sci. USA* **111,** 8470-8475 (2014).

3. Battye, T. G., Kontogiannis, L., Johnson, O., Powell, H. R., Leslie, A. G. iMOSFLM: a new graphical interface for diffraction-image processing with MOSFLM. *Acta Crystallogr. D Biol. Crystallogr*. **67,** 271-281 (2011).

4. Liang, M., et al. The Coherent X-ray Imaging instrument at the Linac Coherent Light Source. *J. Synch. Rad.* **22,** 514-519 (2015).

5. Park, J., Kim, K-H., Nam, B., Kim, S. Current status of the CXI beamline at the PAL-XFEL. *J. Kor. Phys. Soc.* **69**, 1089-1093 (2016).

6. Bostedt, C., et al. Linac Coherent Light Source: The first five years. *Rev. Mod. Phys.* **88,** 015007 (2016).

7. Ko, I. S., et al. Construction and commissioning of PAL-XFEL facility. *Appl. Sci.* **7,** 479 (2017)

8. Barty. A,, et al. *Cheetah*: software for high-throughput reduction and analysis of serial femtosecond X-ray diffraction data. [*J. Appl. Crystallogr*](https://www.ncbi.nlm.nih.gov/pmc/articles/PMC4038800/)*.* **47,** 1118–1131 (2014).

9. White, T. A., et al. *CrystFEL:* a software suite for snapshot serial crystallography. *J. Appl. Cryst.* **45**, 335–341 (2012).

10. Coquelle, A., et al. Raster-scanning serial protein crystallography using micro- and nano-focused synchrotron beams. *Acta Crystallogr*. **D71,** 1184-1196 (2015).

11. Adams P. D., et al. PHENIX: a comprehensive Python-based system for macromolecular structure solution. *Acta Crystallogr D Biol Crystallogr* **66**, 213-221 (2015).

12. Emsley P, Cowtan K. Coot: model-building tools for molecular graphics. *Acta Crystallogr D Biol Crystallogr* **60**, 2126-2132 (2004).

13. Xu, X., Xu, S., Jin, L., Song E Characteristic analysis of Otsu threshold and its applications. *Pattern Recognit Lett* **32**, 956-961 (2011).

14. Haralick, R. M., Sternburg, S. R., Zhuang, X. Image analysis using mathematical morphology. *IEEE Trans Pattern Anal Mach Intell.* **4,** 532-550 (1987).

15. Bezdek, J. C., Ehrlich, R., Full, W. FCM: The fuzzy c-mean clustering algorithm. *Comput & Geosci* **10,** 191-203 (1984).

16. Chapman., H. N., et al. Femtosecond X-ray protein nanocrystallography. *Nature* **470****,**73-77 (2011).

17. Aquila, A., et al. Time-resolved protein nanocrystallography using an X-ray free-electron laser. *Opt. Express* **20,** 2706-2716 (2012).

18. Spence, J. C. H., Weierstall, U., Chapman, H. N. X-ray lasers for structural and dynamic biology. *Rep. Prog. Phys.* **75,** 102601 (2012).

19.  Tenboer, J., et al. Time-resolved serial crystallography captures high-resolution intermediates of photoactive yellow protein. *Science* **346,** 1242–1246 (2014).

**Table S1**. Counting results of microcrystals by experts and the proposed method

|  |  | 1 | 2 | 3 | 4 | 5 | 6 |
| --- | --- | --- | --- | --- | --- | --- | --- |
| Expert 1 | Try 1 | 45 | 161 | 185 | 15 | 60 | 18 |
|  | Try 2 | 45 | 175 | 186 | 15 | 69 | 18 |
|  | Try 3 | 45 | 175 | 186 | 15 | 69 | 18 |
|  | Try 4 | 47 | 173 | 187 | 18 | 61 | 14 |
| Expert 2 | Try 1 | 54 | 151 | 192 | 16 | 83 | 15 |
|  | Try 2 | 54 | 194 | 229 | 16 | 45 | 12 |
|  | Try 3 | 52 | 174 | 200 | 15 | 61 | 15 |
|  | Try 4 | 41 | 169 | 198 | 14 | 54 | 11 |
| Expert 3 | Try 1 | 49 | 157 | 170 | 15 | 45 | 12 |
|  | Try 2 | 38 | 172 | 197 | 17 | 57 | 16 |
|  | Try 3 | 49 | 156 | 157 | 17 | 61 | 16 |
|  | Try 4 | 50 | 149 | 180 | 17 | 68 | 18 |
| Proposed method | Try 1 | 39 | 170 | 189 | 21 | 79 | 16 |
|  | Try 2 | 39 | 170 | 189 | 21 | 79 | 16 |
|  | Try 3 | 39 | 170 | 189 | 21 | 79 | 16 |
|  | Try 4 | 39 | 170 | 189 | 21 | 79 | 16 |

**
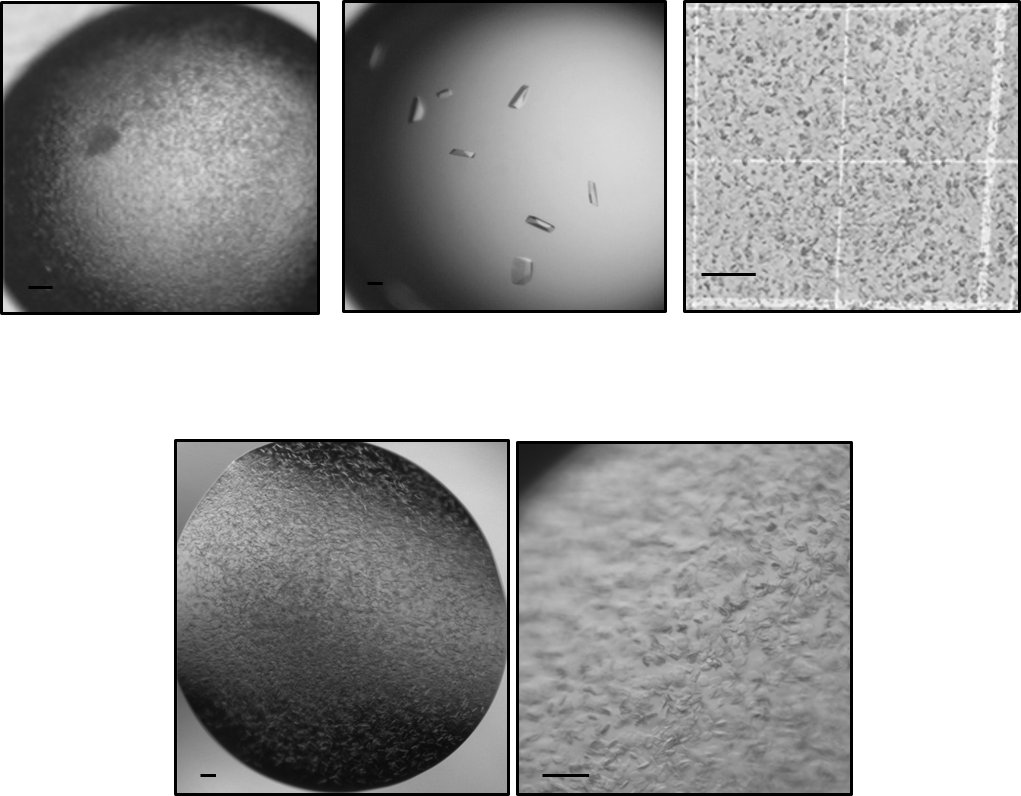
**

**Fig. S1.** **Microcrystallization of HA.** HA microcrystals observed in a vapor diffusion drop by light microscopy. Microcrystallization resulted in occasional formation of granular aggregates, frequent growth to form macrocrystals and the production of showers of tiny micron-sized crystals (upper panel). The microcrystals of HA were detected by ordinary light (left) and bright-field high-resolution (right) microscopy images (lower panel).

**
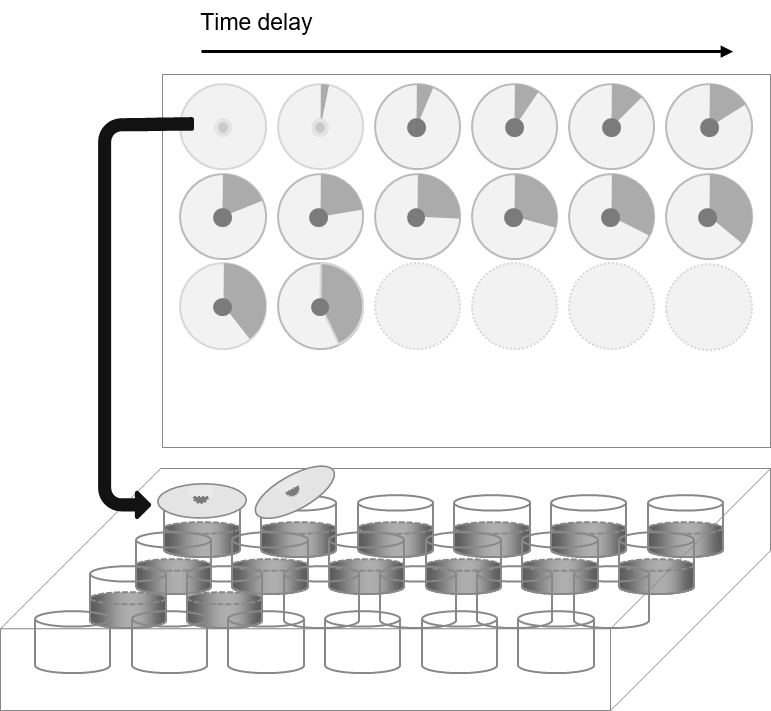
**

**Fig. S2. Diagram of the supersaturation-controlled microcrystallization method.** Partial gray circles around the drops on the coverslips before sealing represent time tracking of sequential evaporation periods.

**
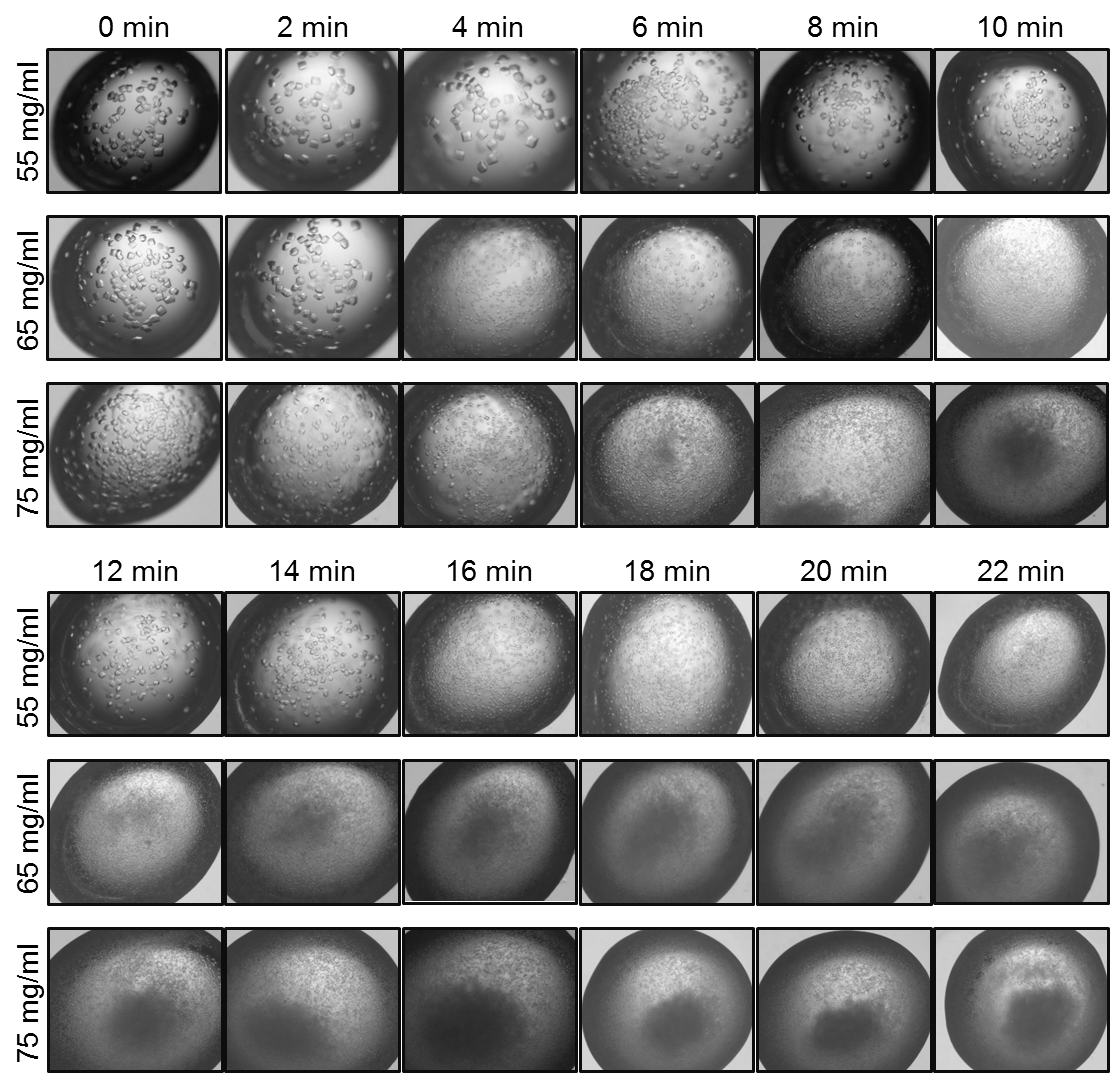
**

**Fig. S3. Images of lysozyme microcrystals**. Hanging drops were obtained by supersaturation-controlled microcrystallization. Mixture of protein and precipitant solutions were dispensed on coverslips which was air-dried until white precipitates start to appear on the first coverslip. The coverslips were then flipped over to seal the wells and microcrystallization was examined by controlling evaporation time from 0 to 22 min with a time delay of 2 min. Smaller microcrystals at higher density were produced with higher concentrations of the lysozyme at the concentrations of 55-75 mg/ml.


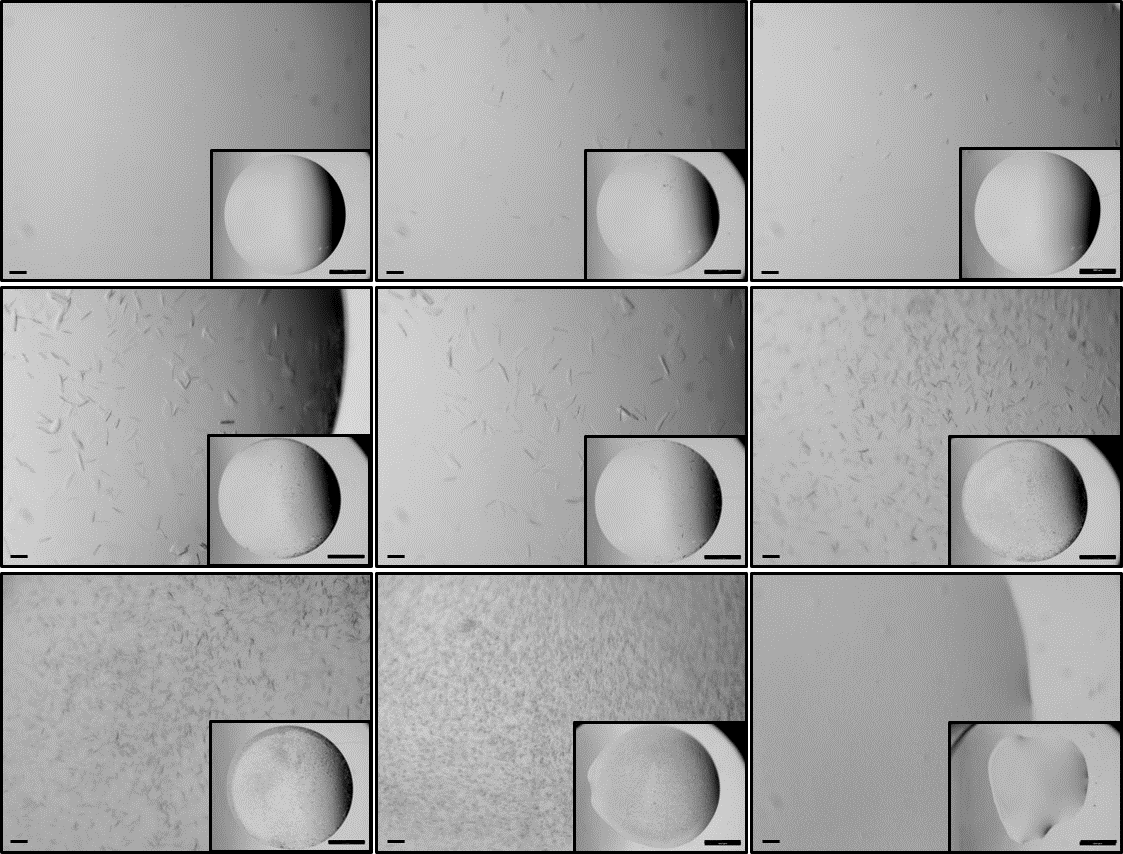


**Fig. S4. Images of HA microcrystals by supersaturation-controlled microcrystallization**. HA microcrystallization was performed for the total duration of 15 min with a time delay of 30 sec and drop images are shown only for the duration of 11-15 min (from the top left corner to the bottom right panels) (Scale bar = 50 µm). Each crystallization drop is shown in the inset. Those at the duration of 14-15 min produced a higher yield of microcrystals.


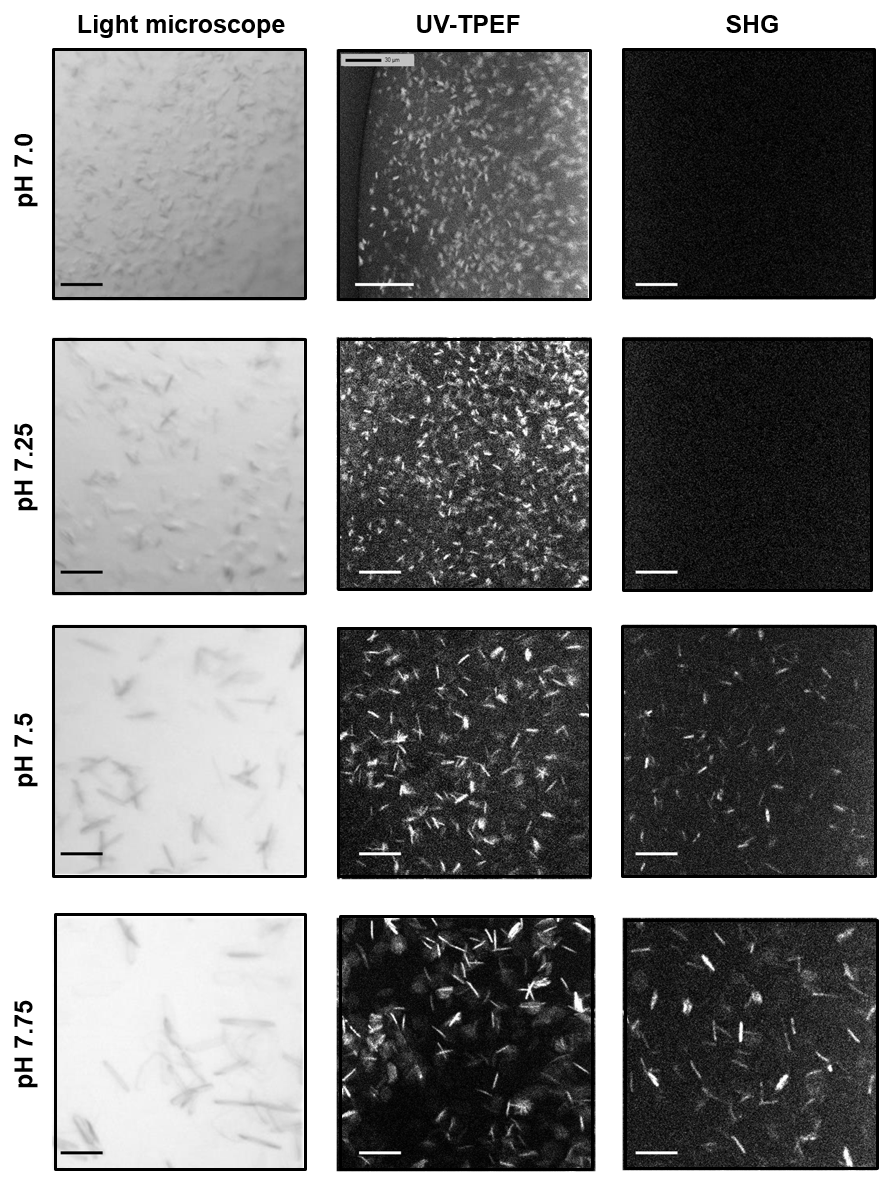


**Fig. S5. Characterization of HA microcrystals**. Microcrystal images of the HA crystals at four different pH conditions detected by bright-field microscopy, UV-TPEF, and SHG. The HA microcrystals showed positive UV-TPEF and second harmonic generation (SHG) signals, except for those obtained at pH 7.0-7.25, possibly due to their small sizes. 1 M Tris-HCl stock solution was used to control the pH of solutions in the range of pH 7.0 to pH 8.0. Scale bars: 50 μm.


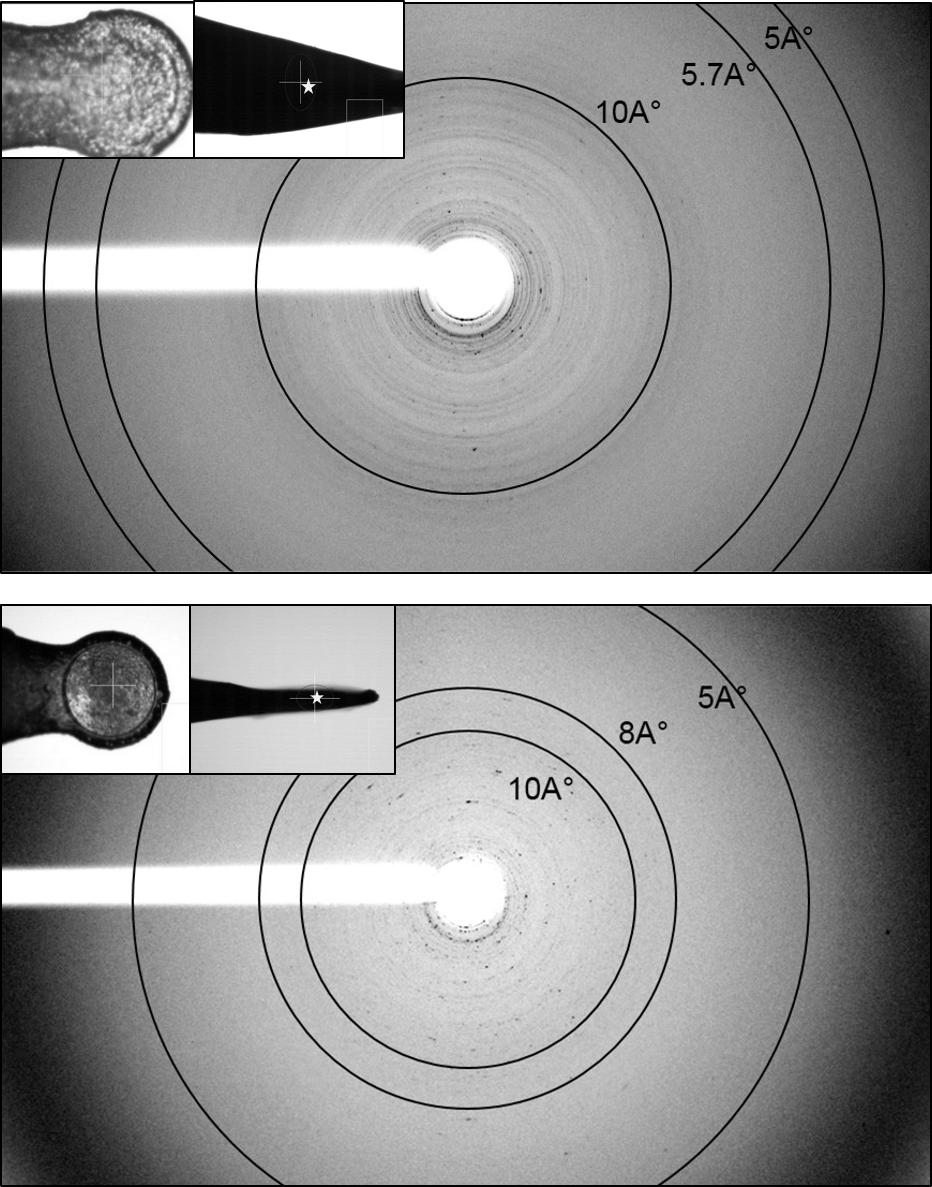


**Fig. S6. Powder diffraction of microcrystals.** **(A)** Images of sample loop (inset left), X-ray beam position (inset right, star) and powder diffraction of microcrystals in 100 mM Tris-HCl (pH 8.5), 30% PEG 400, 200 mM MgCl_2_ (upper panel) and in100 mM Tris-HCl (pH 7.5) and 20% PEG 2000 (lower panel). Microcrystals were spun down to obtain microcrystals at high density in 5% ethylene glycol for cryo-protection and transferred to MiTeGen crystal mounting loop. Powder diffraction with beam size of 200 μm × 110 μm and expose time of 20 sec and the crystal-to-detector distance of 500 mm is shown, indicated by the black ring resolutions.

**
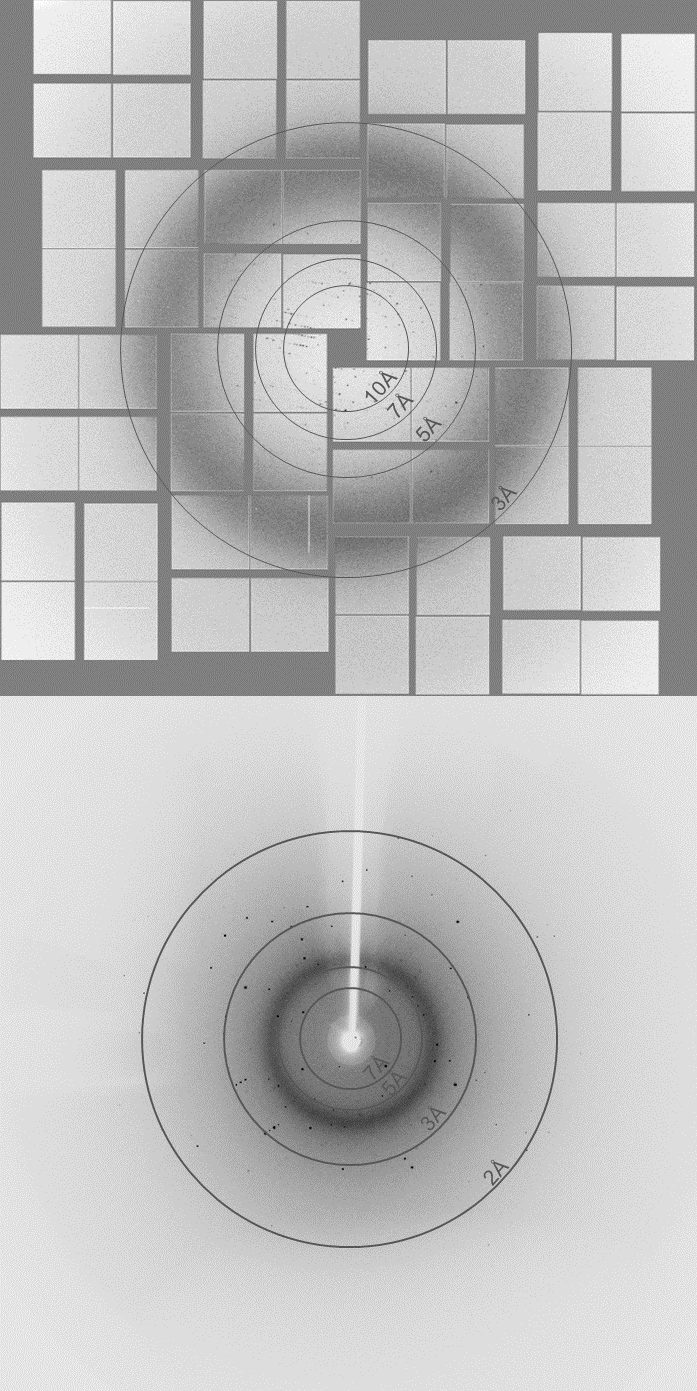
**

**Fig. S7. Single diffraction patterns from HA (upper panel) and lysozyme (lower panel) microcrystals.** Using the CXI instrument of the LCLS and PAL-XFEL, respectively, the diffraction patterns contained Bragg peaks to approximately 3.5 Å and 2.0 Å resolutions, which were indexed with unit cell parameters very similar to those obtained from single crystals.


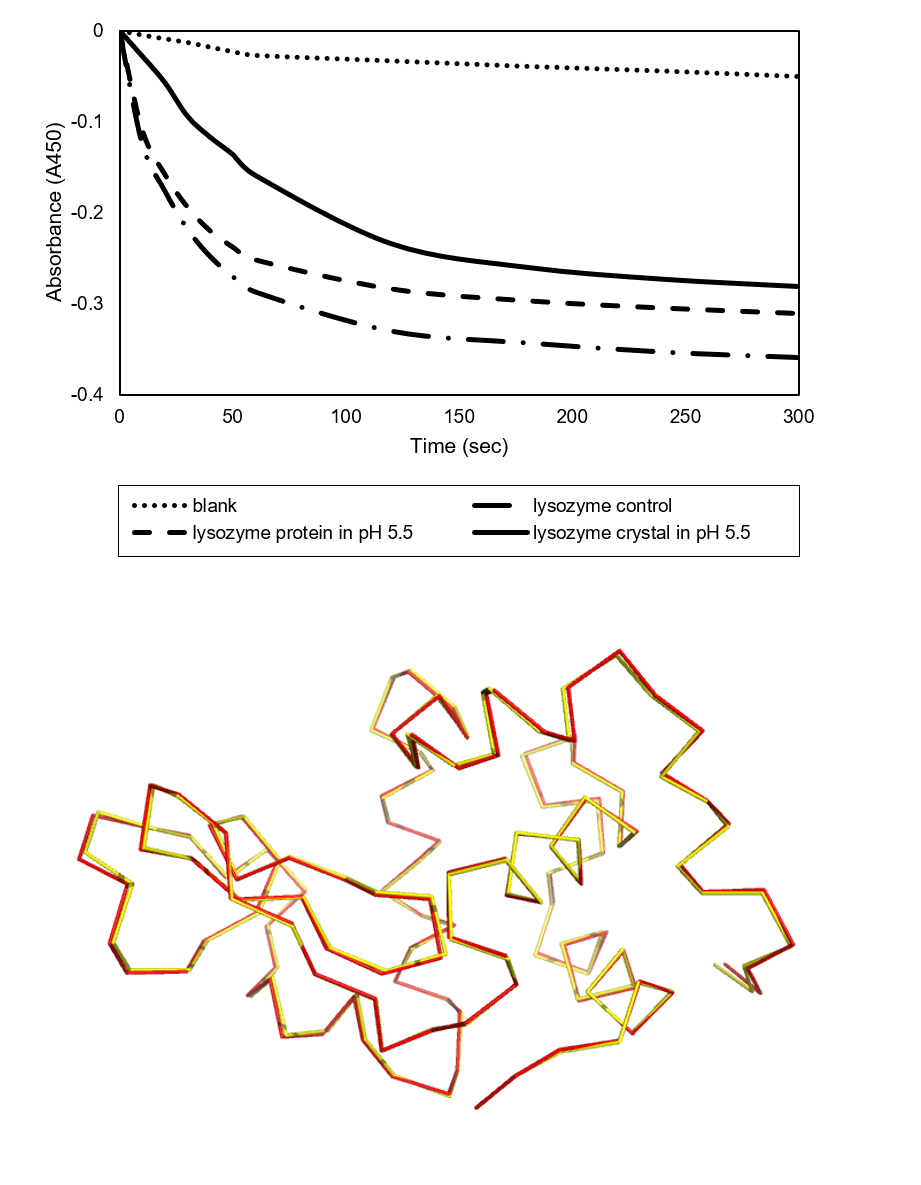


**Fig. S8. Enzyme activity and tertiary structures of lysozyme.** Dissolved lysozyme microcrystals showed enzyme activity at pH 5.5 (upper panel), and superposition of the refined structure of lysozyme (red color) with that of PDB entry 1H6M (yellow color), showing very similar tertiary structures (lower panel).


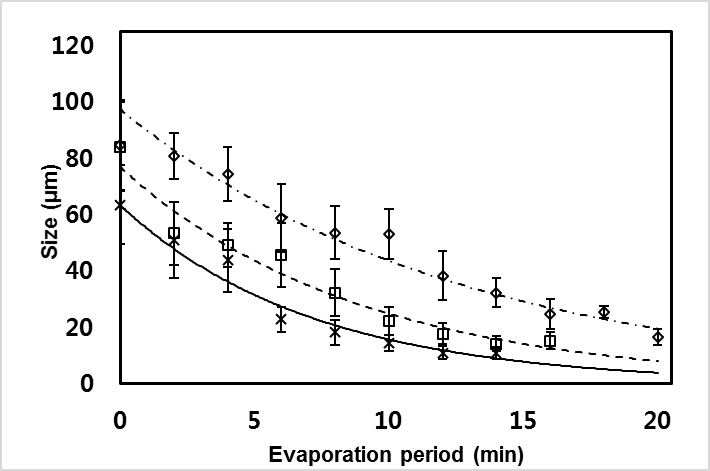


**75 mg/mL**

**65 mg/mL**

**55 mg/mL mg/ml7**


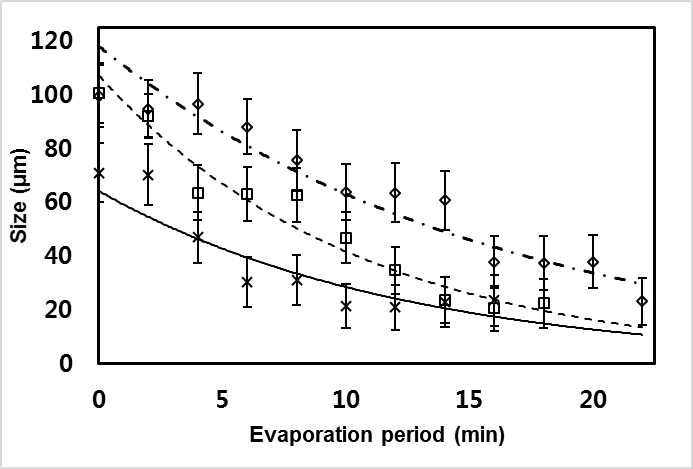


**Fig. S9. Plots of microcrystal size as a function of evaporation period for microcrystallization of lysozyme**. The microcrystal sizes were analyzed manually (upper panel) and by the proposed algorithm (lower panel). Dashed dotted, dashed, and continuous lines represent lysozymes at 55, 65, 75 mg/mL, respectively. The lines are for a 1.8 μL drop with evaporation period from 0 to 22 min. The data represent the means±SD.

**Fig. S10. Plots of microcrystal size as a function of evaporation period for microcrystallization of ferritin**. The microcrystal sizes were analyzed manually (dashed double dotted line) and by the proposed algorithm (continuous line). The lines are for a 1.8 μL drop with evaporation period from 0 to 27 min. The data represent the means±SD.


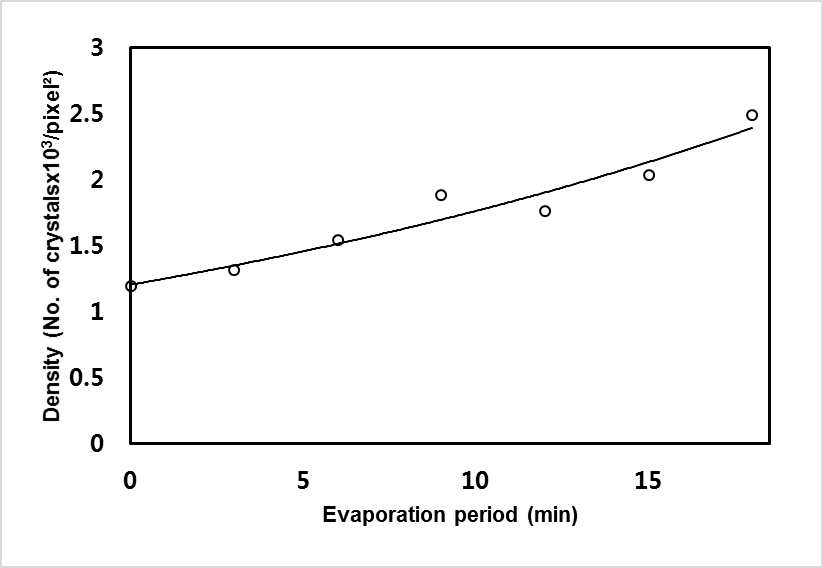

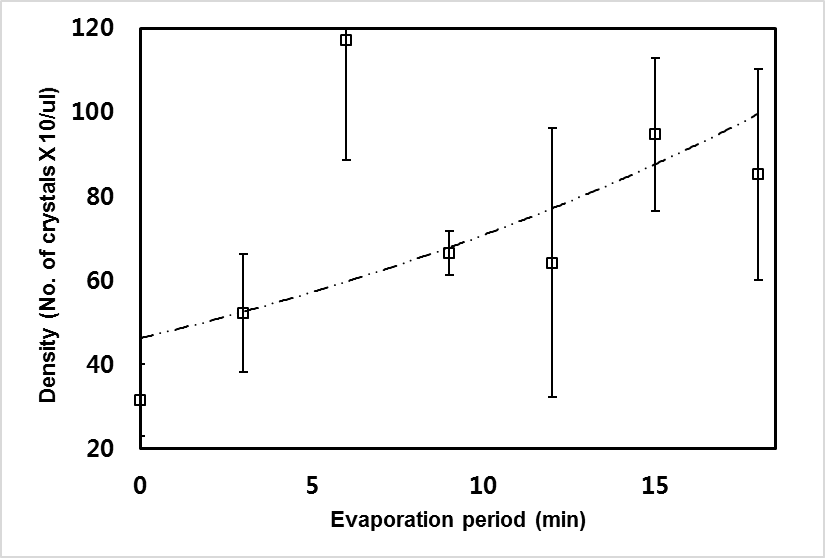


**Fig. S11. Plots of microcrystal density as a function of evaporation period for microcrystallization of ferritin**. The microcrystal densities were analyzed manually (upper panel) and by the proposed algorithm (lower panel). The lines are for a 1.8 μL drop with evaporation period from 0 to 18 min. The data represent the means±SD.
